# Clarifying Some Fundamental Errors in Herries' “A Chronological Perspective on the Acheulian and Its Transition to the Middle Stone Age in Southern Africa: The Question of the Fauresmith” (2011)

**DOI:** 10.1155/2012/230156

**Published:** 2012-05-10

**Authors:** Lawrence Barham

**Affiliations:** Department of Archaeology, Classics and Egyptology, University of Liverpool, Hartley Building, Liverpool L69 3GS, UK

## Abstract

Herries provides a timely review of the archaeological and dating evidence of the transition from the Acheulean to the Middle Stone Age (MSA) in southern Africa, however, in relation to the site of Twin Rivers, Zambia he makes several fundamental errors of interpretation that demand correction. The stratigraphic sequence of the site is admittedly complex, but it deserves a more careful analysis than that offered by Herries. This detailed response by the most recent excavator of the site addresses Herries critique by placing the site in its historical context and then dealing with the central issue of the association of dated speleothem with the surviving archaeological deposits. Herries is shown to have mistakenly combined the dates from two separate cave passages and to have misunderstood the published sections, plans, and taphonomic assessment of each excavation area. His reinterpretation of the site as being significantly younger than published is based on a conflation of unrelated data.

## 1. The Twin Rivers Review

Herries ([1, page 11] ) reanalyses the published data from the most recent excavations at Twin Rivers and argues that there is considerable uncertainty about the age of the deposits and as a consequence about the significance of the site. He concludes that the sequence may be significantly younger than claimed and makes the general observation that “Excavators need to be extremely careful when relating fragments of flowstone to wider archaeological deposits in caves due to their complex depositional history…. Often dates are presented without any information regarding their reliability or context” ([1, page 11]). As the archaeologist responsible for the most recent research at Twin Rivers, I concur with these comments. Herries, however, in the case of Twin Rivers has misunderstood the stratigraphic sequence of the site and unwittingly conflated dates from unrelated deposits. He has also overlooked our critical assessment of the reliability of the association of the dated material with the archaeological deposits. His resulting reinterpretation throws considerable doubt on the integrity of the published association of dates with the deposits excavated in 1999. According to Herries his concerns are shared more widely: “….many researchers are sceptical over the association of the flowstone to the MSA bearing deposits.” If this is indeed the case [no sources are cited] then this uncertainty needs to be discussed in detail so that the reader can assess the validity of the critique. A brief history of the excavations provides the context for understanding the limitations of the site and the rationale for the most recent research in the 1990s. The location of the dated samples in relation to the excavated deposits is made clear which in turn reveals the source of Herries' confusion.

## 2. History of Excavation: F, A, and G Blocks (1954–1999)

Twin Rivers hill is located near the capital city of Lusaka and was extensively excavated in 1954 and 1956 by Clark [2]. The excavated deposits were bone and artefact bearing breccias found in karst features (fissures and former cave passages) on the top of the hill and down its western flank. The largest area excavated was labelled F Block (on the hill top) and below it the breccias were sampled in a series of excavation blocks labelled A–E. The breccias were removed by controlled blasting using dynamite, and the blocks were reduced manually to extract artefacts. The artefacts were attributed to the Lupemban Industry of the Middle Stone Age based on similarities with Lupemban material excavated at Kalambo Falls to the northeast [3]. Radiocarbon dating was the only relevant radiometric technique available in the 1950s, and unsurprisingly the lateral extent of flowstone deposits was not recorded as that information was not considered significant at the time. (The development of uranium-series dating of carbonates in the 1970s postdated the excavations at Twin Rivers.) It was clear from Clark's [2] published sections that flowstone was interlayered with the F Block breccia. The lack of information on the lateral extent of flowstone remains problematical for reconstructing the association of the dated flowstone with the breccia deposits. The roofs of the cave passages (F and A Block) had collapsed in the past and so it was not possible to identify points of entry for water that formed the flowstone deposits. The hill surface had also undergone extensive lowering by dissolution that meant the original height and size of these two separate caves could not be reconstructed (see [4], pages 169–172 for site formation details). These far from ideal conditions were the starting point for the research undertaken in the 1990s.

The site was reexamined in 1995 to collect flowstone from F Block for uranium-series dating (^230^Th/^234^U). A band of flowstone illustrated in Clark's section drawing was sampled from near the top of the 1956 excavation, but only isolated patches of breccia remained on the passage surfaces as recorded in our Figure  10.15 ([4, page 182]). The Middle Pleistocene age that resulted (230 ± 35/28 ka [5], Barham and Smart 1996) and subsequently redated using higher precision thermal ionisation mass spectrometry [TIMS] to 195 ± 19 ka, [6] indicated that the underlying Lupemban was probably of comparable age if not older. Although no breccia was sampled, we found a small tufa and sediment filled dip tube off the passage, and that material was excavated in 1999. These deposits could not be associated directly with dated flowstone from the passage. In 1999, a flowstone was found that extended from near the base of the passage to the top and it was sampled at its thickest point (overlying a limestone shelf) for dating. The resulting two ages from different layers within the sample were younger (~178 ka and ~139 ka, see below) than the flowstone from near the top of Clark's section. As we reported, the irregular morphology of the F Block passage had made the stratification of speleothem more complex than in A Block ([4, page 181]).

Down slope in A Block sufficient breccia deposits survived along the western wall of this passage to justify making this area the focus of excavation in 1999. A series of flowstone deposits also survived adhering to the walls and floor of the A Block passage, and one sample was interlayered with the artefact bearing breccia. This flowstone was the basis for the ~266 ka date for the deposit. The underlying breccia contained a fragment of flowstone dated to beyond the age limit of the technique (>400 ka), and a flowstone on the floor of the cave passage was also beyond the age range (>400 ka). Above the breccia were two separate flowstones that formed in either a passage or fissure now destroyed by the erosion of the hill slope ([4, page 178]). These flowstones form part of the concordant sequence of dates from this western wall (below), and more significantly they provided a minimum age for the underlying archaeological deposit of 172 ka/225 ka. The A Block dates are discussed in more detail below as they are critical to unravelling the source of Herries' confusion, but there is one more area of the site to be described in this history as it too features in his reinterpretation of the site's chronology.

G Block forms a part of the hilltop platform and is separate from F and A Block and is not a cave or fissure but a lag deposit resulting from the dissolution of the limestone. Though not part of the original project plan for 1999, the sampling of G Block was initiated to give a better understanding of the archaeological record on the surface of the hill top and to assess its formation. Clark found Later Stone Age and Iron Age material in these surface deposits, and our excavation confirmed his observations but also added Middle Stone Age tools to the record. The densest concentrations were found in a shallow linear root-filled solution feature (grike) from which burnt quartz and sediments were collected for thermoluminescence (TL) dating ([4, pages 181–183]). As the excavation of G Block had not been planned in advance, there was no opportunity to incorporate *in situ* dosimetry as part of the sampling procedure. The resulting TL dates were all Late Pleistocene in age (ranging from ~101 ka to ~13 ka), but they showed no consistency with depth with the youngest dates associated with the base of the grike ([4, page 183]). Given the extent of mixing, no quantitative analysis of the archaeological deposit was undertaken. Our report included illustrations of Middle and Later Stone Age retouched tools to demonstrate their presence, and the suggestion was made that Middle Stone Age might be associated with the ~101 ka date.

Herries unfortunately incorporated the G Block TL dates in his analysis of the F and A Block speleothem dates without making reference to our published assessment of the deposits as being mixed. He inserted the misleading comment that the G Block dates, being younger than the speleothem ages, “lend further suspicion to an extremely complicated stratigraphy and infill” (the full quote is presented below). The G Block sediments are of course part of Twin River's archaeological record, but they are unrelated to the formation or age-range of the cave passages and should be treated with great caution generally.

## 3. A and F Block Dates in Detail

The primary area of excavation in 1999 was the surviving breccia and decalcified sediments adhering to the western wall of the A Block passage. The main aim of the excavation was to recover artefacts from contexts that could be dated by association with speleothem (using U-series TIMS). Six flowstone samples were collected from A Block of which five came from the western wall of the passage and the sixth (TRA5A) was from the eastern side of the passage and unrelated to the surviving deposits as was stated clearly and illustrated in our report ([4, pages 178, 181 and Figure  10.5]). With the exception of one sample (TRA14A) all the flowstone samples were collected from lenses overlying and within the breccia ([4, page 172]). TRA14A was unusual in that it was a fragment of flowstone that had become incorporated into the breccia. We included a photograph (Figure  10.14) showing the stratigraphic context of this one dislocated piece of flowstone as part of our critical discussion of the dating of A Block ([4, page 178]). Photographs of the intact flowstones were not included as they were the norm in both A and F Block, but Herries' point is well taken that photographs should have been included for each speleothem. He also suggests that micromorphological analysis of the contact between the speleothem and archaeological deposit would help assess the depositional history of each sample. This is considered good practice now, but as far as I am aware it was not the norm in 1999. Our section drawings (10.13 for A Block, 10.15 for F Block) show the vertical location of the dated samples associated with the surviving breccias and the plan of A Block (Figure  10.5) shows the horizontal location.

In A Block, only two samples were accepted by us as being in contact with the archaeological deposits, TRAA1 and the fragment enclosed in breccia, TRA14A. TRAA1 is a flowstone lens at the base of breccia wedged between two limestone blocks. The breccia grades into a decalcified deposit (red sandy earth, Figure  10.13) that is preserved between the passage wall and the adhering breccia that forms a continuous deposit along the western wall. The detritally corrected age of the flowstone (266 ka) was interpreted as representative of the breccia deposits at approximately this depth in the absence of other dating controls. (The one TL date on calcite from the decalcified deposit lacks *in situ *dosimetry, and as Herries' correctly observes it cannot be considered to be reliable.) The remaining flowstone samples provided maximum and minimum ages for the existence of the cave and for the archaeological deposits excavated in 1999 ([4, page 172]).

Samples TRA4A and TRA3A both formed in the passage or fissure *above* the breccia (Figure  10.5) and each was sub-sampled for dating as two distinct layers could be seen ([4], page 175). Below the 266 ka age, the flowstone fragment incorporated into the breccia (TRA14A) exceeds the age range of the U-series technique used and a sample of flowstone adhering to the bedrock below (TRA2A) also has an open date of >400 ka. We speculated that this basal flowstone might have been the source of the redeposited fragment but concluded that these two samples only provide a potential maximum age for the archaeological deposits ([4, page 178]). We do not know how long after the formation of the basal flowstone that sediments began to fill the passage as slurry flows. There is a hiatus but of unknown duration. The deposits may all be 266 ka and younger or perhaps slightly older given the depth of deposit below TRAA1 [7, 8].

The age determinations for each speleothem in A and F Block follow and include the uncorrected age, sample identifier, depth below datum, and the corrected age (for further analytical detail see Barham et al. 2000 [4, Table  10.1]). The thick flowstone sample from F Block (Figure  10.15, east wall) was subsampled and the results are presented for each layer as is the case for TRA4A and TRA3A in A Block. The corrected ages are included as these have been used in other publications [9, 10].

**A Block, Western Wall:**

173 ± 3 ka (TRA4A, 243 cm, layer 1, corrected age = 173 ka),
166 ± 3 ka (TRA4A, 243 cm, layer 2, corrected age = 160 ka),
225 ± 4 ka (TRA3A, 320 cm, layer 1, corrected age = 225 ka),
178 ± 2 ka (TRA3A, 320 cm, layer 2, corrected age = 172 ka),
275 ± 6 ka (TRAA1, 340 cm, corrected age = 266 ka),
>400 ka (TRA14A, 383 cm),
>400 ka (TRA2A, 390 cm).

**A Block, Eastern Wall:**

199 ± 2 ka (TRA5A, 220 cm, corrected age = 192 ka).

**F Block, Eastern Wall:**

181 ± 6 ka (TRF layer 1, 163 cm, corrected age = 178 ka),
141 ± 2 ka (TRF layer 2, 163 cm, corrected age = 139 ka).

## 4. Herries' Reinterpretation

The above history of the excavation has been presented in some detail to address Herries' claim that “In many instances, flowstone is sampled from the wall or edges of a cave cavity without definitive evidence for its association to the archaeology”. I repeat, only two sample of flowstone were considered by us as being in contact with the archaeological deposits, TRAA1 and TRA14A, and both were from A Block. The near absence of continuous layers of flowstone that could be linked directly to the deposits is an artefact of the history of excavation. The following extended quote from Herries ([1, page 11]) contains the primary source of confusion in his account, and the points of misunderstanding are labelled with capital letters for referencing in the discussion that follows.

 “(A) The fact that younger speleothem dated to between 184–172 ka (178 ± 6 ka) and 141–137 ka (139 ± 2 ka) occurs under a speleothem dated to between 200–190 ka (195 ± 5 ka; 131) in block A. [*sic*] All TL dates from G block are younger than 117 ka (101 ± 16 ka) and lend further suspicion to an extremely complicated stratigraphy and infill. (B) The speleothem dates to between 266 ± 6 ka (272–260 ka) and 172 ± 2 ka (174–170 ka) may also have been eroded out from earlier deposits when the MSA in-filled the cavity. (C) Again younger speleothem samples occur with depth with the 172 ka sample being deeper (3.2 m) than the 192 ± 2 ka sample at the top of A Block at 2.2 m. (D) All the MSA in the top 1 m of A block is, therefore, younger than 174 ka, as the speleothem must have formed before it was eroded and incorporated into the breccia and so provides a maximum age.”

- There is no speleothem dated to 141–137 ka (139 ± 2 ka) in A Block. The cited age range most closely matches sample TRF (layer 2, 141 ± 2 ka uncorrected, 139 ka corrected) from *F Block* (see above). The sample that dated to 195 ± 5 ka (TRA5A) occurs on the eastern wall of the A Block passage, and unless Herries assumes that it was once continuous across the passage, it cannot be related to the west wall speleothem. That would be bad practice. This spatial separation was made clear in the published report and seems to have been overlooked.
- The speleothem is flowstone deposits that are *in situ* as described in the report (above) and not redeposited fragments. Only sample TRA14A (>400 ka, 383 cm) is a redeposited fragment of speleothem as was stated clearly in the report ([4, page 178]). Herries expands this line of reasoning later to suggest the “If the majority of speleothem represents material eroded into the deposit then the Lupemban from block A would be younger than 141 ka, significantly younger than the 266–170 ka cited by Barham et al.” The 141 ka date is erroneous in relation to A Block as is the suggestion that the majority of the speleothem samples are redeposited.
- Again, the sample dated to 195 ± 5 ka (TRA5A) cannot be related to any other speleothem on the western wall of A Block. This is a fundamental error of attribution.
- There is no extant Middle Stone Age material in the upper 1 m of A Block, only from below sample TRA3A (320 cm) (see [4, page 178 and Figure  10.13]).

Herries continues this line of reasoning later in stating that “The sample with a date of 160 ± 3 ka also occurs at 2.4 m depth and is the youngest age from block A. This suggests that all the MSA in block A may in fact be younger than 163 ka.” This sample (TRA4A, layer) overlies TRA43 and as described clearly in our report is related (along with TRA3A) to a separate passage or fissure *above* the archaeological deposits and unrelated to the material excavated in 1999.

## 5. Conclusion

Science-based dating underpins our understanding of the process of human evolution and its variability in place and time. The past 20 years or so have seen a revolution in the development of radiometric methods of dating that have opened the Middle Pleistocene to closer and more informed scrutiny by palaeoanthropologists [11]. We can now see more clearly the tempo of changes in human anatomy and behaviour that were previously obscured by poor chronological controls [12]. It is in this context of an improving database that Herries' [1] provides a much needed review of the archaeological and dating evidence of the transition from the Acheulean to the Middle Stone Age in southern Africa. Thoughtful reviews play an important role in the development of a discipline by distilling what is known about a subject and highlighting issues for further investigation. Reviews are also often the first port of call for non-specialists and advanced students in need of a clear statement of the state of the art of a subject. Herries cavalier treatment of the Twin Rivers published data, however, undermines my confidence in what appears otherwise to be a comprehensive treatment of the Acheulean-Middle Stone Age transition.

Twin Rivers remains an important Middle Pleistocene site despite its history of excavation and complex stratigraphic sequence. I stand by the published interpretation of the age range of the limited A Block deposits and am actively searching for a “new” Twin Rivers that can be excavated to the highest of current standards. This is the way forward in a region for which we still know so little about the behaviour of hominins before the evolution of *Homo sapiens*.
